# Sensing Applications in Aircrafts Using Polymer Optical Fibres

**DOI:** 10.3390/s21113605

**Published:** 2021-05-21

**Authors:** Pedro C. Lallana, Gotzon Aldabaldetreku, Alicia López, David S. Montero, Gaizka Durana, Javier Mateo, M. Ángeles Losada, Joseba Zubia, Carmen Vázquez

**Affiliations:** 1 Electronics Technology Department, Universidad Carlos III de Madrid, E-28911 Leganés, Spain; dsmontero@ing.uc3m.es; 2 Communications Engineering Department, University of the Basque Country (UPV/EHU), Ingeniero Torres Quevedo 1, E-48013 Bilbao, Spain; gotzon.aldabaldetreku@ehu.eus (G.A.); gaizka.durana@ehu.eus (G.D.); joseba.zubia@ehu.eus (J.Z.); 3 GTF, Aragon Institute of Engineering Research (i3A), University of Zaragoza, María de Luna 1, E-50018 Zaragoza, Spain; aliclope@unizar.es (A.L.); jmateo@unizar.es (J.M.); alosada@unizar.es (M.Á.L.)

**Keywords:** fibre sensors, temperature measurement, polymer optical fibre, micro-vibrations, level, strain, Power-over-Fibre (PoF) connectors

## Abstract

We report on recent advances in the use of inexpensive polymer optical fibres (POFs) for sensing applications in avionics. The sensors analysed in this manuscript take advantage of the unique properties of polymers, such as high flexibility, elasticity, and sensitivity, and they range from strain, elongation, and vibration interrogators to level and temperature meters, leading to cost-effective techniques for structural health monitoring in aircraft structures. We also highlight recent power-supply methods using Power-over-POF in order to feed sensors remotely, and we discuss the constraints imposed by connectors on the performance of POF networks in aircrafts.

## 1. Introduction

The gradual implementation of the “fly-by-wire” concept has greatly reduced the complexity and weight of traditional mechanical systems, also increasing the safety and stability in aircrafts [1]. As a result, typical commercial or military aircrafts consist of a number of control and monitoring systems that demand a large number of sensors distributed along the airplane and whose output electrical signals are routed through a copper cable network to the computers that command the corresponding actuators. Over the last decades, the growing climate concerns have moved the avionics industry to reduce emissions and improve fuel efficiency by the introduction of composite fuselages to substitute the conventional metallic materials, thereby building lighter aircraft models. Although this critical change also entails a lowering of the manufacturing and operating costs, composite structures subject avionic systems wiring to a more severe level of lightning-induced voltage and current. In fact, the consequent electrical wire damage is one of the major factors leading to airplane failures [2]. In this context, the high electromagnetic immunity of optical technologies has promoted the migration of copper harness to fibre-based systems following the “fly-by-light” concept [3]. More recently, wireless avionic networks have been brought under consideration, but assurance, reliability and security concerns may restrict their use to a set of specific non-critical tasks [4]. In addition, an ultra-modern aircraft has higher operational needs motivated by the increase in safety-critical systems for flight and engine control, as well as non-safety-critical systems, such as structural and Engine Health Monitoring (EHM) systems, cabin environmental control systems, etc., which can be better answered in the optical domain [5,6,7].

Fibre optic technologies benefit from optical fibre electromagnetic interference (EMI) immunity, electrical passiveness, and fire-hazard resistance, which are indispensable to develop sensing devices and systems for harsh environments, such as aircrafts [8]. In addition to their small package size and lightweight, fibre optic sensors (FOSs) have increased sensitivity and lower power consumption compared to other sensing technologies. Their geometric versatility permits deployment with arbitrary shapes and embedding into structures. Moreover, FOSs are compatible with fibre-based broadband communications systems and have the capacity to carry out remote sensing over long distances. This feature is particularly interesting for airplane monitoring systems whose data need to be transmitted with stringent bandwidth requirements to central repositories for data processing applications and with low latency for control-related signals [9]. The fibre optic potential for multiplexing introduces the added advantage of developing Power-over-Fibre (PoF) strategies to feed the sensors and access control systems remotely [10].

On the other hand, these advantages of FOSs are partially counterbalanced with the inherent installation and maintenance costs that are particularly high for glass optical fibres (GOFs). As glass fibres are mechanically weak and generally lack bending ability, they are susceptible to breakage and contamination at the assembly and installation stages, and have to be handled with care. In addition, in a typical aircraft deployment, many in-line connectors are needed for production breaks. Connection insertion for GOFs is a very demanding process even for multimode fibres (MMFs), whose core diameters are larger (up to 62.5 µm) than the 9 µm of standard single-mode fibres. Thus, the termination of the fibre end-face and precision alignment have to be performed by experts or highly trained operatives. Most of these requirements, however, can be relaxed if polymer optical fibres (POFs) are used instead of glass fibres.

The term POF encompasses a variety of fibres whose core and cladding are made of plastic materials: poly(methyl methacrylate) (PMMA) and perfluorinated (PF) polymer [11] are the most common. PMMA has so far been the primary thermoplastic material for POFs. It is a widely used material in many industries under several different trade names, such as Perspex, Plexiglas, Crylux, and Acrylite. In addition to its excellent optical quality and high flexibility, PMMA has other good properties, e.g., processability, good resistance to alkalis, aqueous inorganic salts and dilute acids, hydrolysis, and UV-induced aging. PF polymer fibres have smaller core diameters (50 to 250 µm), and their transmission performance can even surpass that of multimode GOFs [12]. In contrast, the PMMA fibres have larger core diameters (normally 900 µm) and numerical apertures from 0.3 to 0.6. They are manufactured with a different number of cores and with various index profiles: the step-index (SI), multi-step index (MSI), or graded index (GI) profile [13,14]. Microstructured POFs (mPOFs), the analogue version of the photonic crystal fibres with polymer fibres instead of glass fibres, can also be designed and drawn [15,16]. Interesting types of polymers for mPOF manufacturing are the cyclic olefin copolymers (TOPAS), which is regarded as the best choice for humidity insensitive fibre-optic sensors (FOSs); the cyclo-olefin homopolymer (ZEONEX), which not only is humidity insensitive but also has a high glass transition temperature [17]; polycarbonate (PC), with excellent clarity and impact strength properties [18]; and finally, fluorinated polymer (CYTOP), which present the advantage of longer operational distances, such as its low-loss at the 1550 nm region [19]. However, the most important disadvantage of using these mPOFs reported so far in the literature is their lack of connectivity with the world away from controlled laboratory environments, without an optimized method yet for their end-face termination compared to standard PMMA SI-POFs.

In general, large core PMMA POFs have narrower bandwidths (40 MHz for 100 m) and higher attenuation (0.11–0.25 dB/m at 650 nm) than glass fibres, but they are still adequate to meet the demands of short-range networks in automotive, industrial, domestic, and, more recently, avionics environments [20,21,22,23]. Moreover, special large-core PMMA fibres with thermoset polymer jackets have become commercially available in the last years [24,25,26]. Unlike other polymer and most glass fibres, these POFs can sustain temperatures up to 105 °C and thus are capable of fulfilling the firm environmental requirements for the aircraft [27]. Therefore, large-core thermoset SI-POFs have good potential for avionics data systems and networks where the majority of links are short and low speed, although their high attenuation and connectivity pose a challenge for the optical power budget [28].

Added to the advantages shared with GOFs of EMI immunity and electric passiveness, there are other specific characteristics that make POFs very attractive to the avionics industry. The PMMA has a lower density than silica, resulting in even lighter optical fibres [20] and it is resistant to impacts and vibrations. In general, POFs are very flexible and have relatively low curvature losses, which are very good traits for spreading to all areas of the aircraft. Their larger core diameters and higher numerical apertures enable easy connections with other devices using inexpensive connectors and avoiding the need for expensive termination tools. Consequently, POFs are very easy to manipulate and do not require expert handling, which reduces the cost of deployment and maintenance. In fact, this “do-it-yourself” feature is behind the success of POFs in home applications. Moreover, PMMA fibres have attenuation minima in the visible range of the spectrum (530, 570, and 650 nm), an obvious advantage for failure detection and safety issues. In addition, the POFs’ large diameters endow them with higher tolerance to vibrations and to light blocking by dust particles, both important issues in airplane environments. Therefore, POFs can be used with cost-effective active and passive components to build versatile POF-based systems [29].

Specifically, as the base of the sensing devices, POFs have the advantages common to all MMFs, whose great flexibility and small size enable a good compromise between sensitivity and volume. The POFs’ unique assets to develop sensors are their high elastic strain limits, high fracture toughness, high flexibility in bending, high sensitivity to strain, and potential negative thermo-optic coefficients [30]. In addition, their higher numerical apertures and larger diameters optimize their interaction with the medium to be measured. It has been proved that a POF can be used to sense a great variety of parameters, including temperature, humidity, pressure, organic and inorganic compounds, wind speed, and refractive index. The most frequent sensing technique for POFs is the detection of an intensity variation, but path difference, Fibre Bragg Gratings (FBGs), and interferometry (including the speckle analysis of the light pattern distribution at the POF fibre end face after propagating) have also been used to develop sensors in many application fields [31].

In this paper, we are going to focus on the subset of POF-based sensors and applications that have potential use in the avionics industry, including those already developed and presently in use. The following section reports on strain-sensing techniques based on POFs and applied to Structural Health Monitoring (SHM). The third section describes the POF sensors for EHM and introduces a novel technique for remote sensing of micro-vibrations. The fourth section deals with a self-referencing intensity-based POF sensor for liquid detection and discusses its usage in harsh environments, such as oil/petrol tanks in the aircraft. The fifth section focuses on the design and development of a POF macrobend temperature sensor that can be applied to the crucial temperature control in some parts of the aircraft. The sixth section evaluates POF links as auxiliary technologies for remote power distribution through a power-supply method using Power-over-POF techniques, and the impact of air-gap connectors in a POF-based data network. To conclude, we offer a summary of the achievements of POF sensing in the avionics industry.

## 2. Strain Measurement in Aircraft Elements

SHM is essential in aircraft transportation to reduce the time and costs related to maintenance. It also allows a constant survey of the structures subjected to strain in order to avoid too high values for safety reasons. For such a purpose, many researchers have reported on different proposals using different types of optical fibres. However, POFs stand out as ideal candidates for the measurement of high strains values thanks to their greater Young’s modulus.

**Figure 1:**
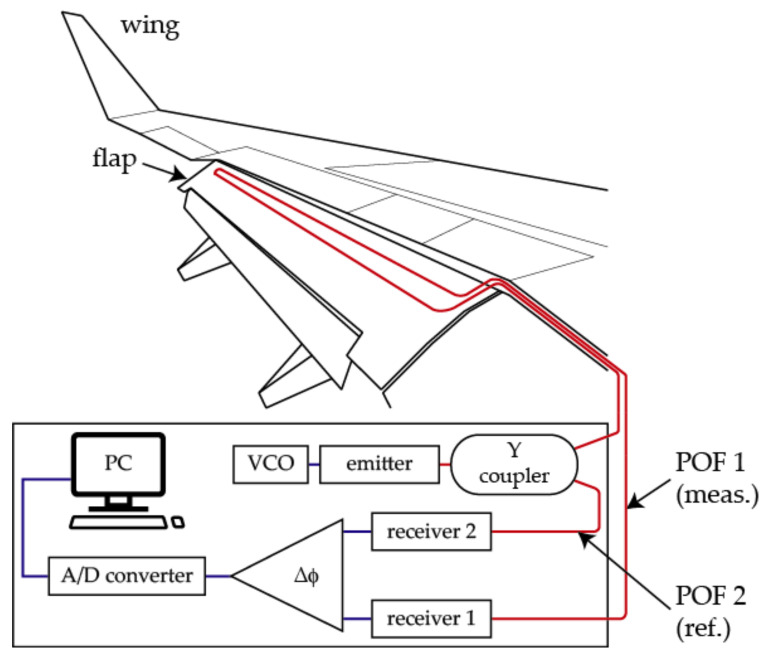
Schematic of the POF elongation sensor to measure the elongation of an aircraft flap. A voltage-controlled oscillator (VCO) modulates the light signal. Δφ denotes the phase comparator.

A POF elongation sensor can be useful to measure the elongation of an aircraft flap, as reported in [32,33]. Figure 1 shows the schematic of the set-up and its working principle. It relies on the comparison of the phase difference between two modulated light signals: a variation due to an elongation of one of the POFs (POF 1) leads to a phase shift in the signal arriving to the corresponding photodetector (Receiver 1).

**Figure 2:**
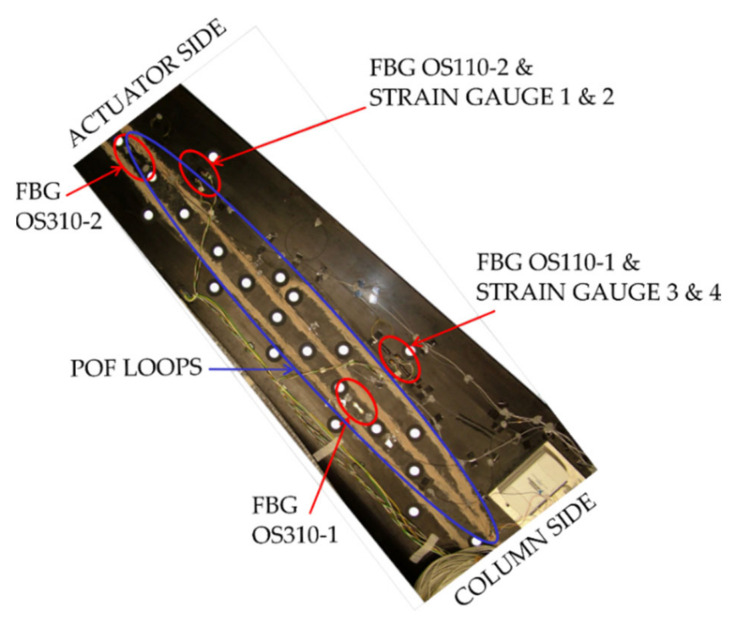
Upper skin of an elevator from a commercial airplane with the sensors bonded to its surface.

Tests for this sensor have been carried out on the upper skin of an airplane elevator made of a carbon-reinforced composite with a honeycomb core. In order to attach the POF sensors and the electronics, the authors of the reported work have used two different glues, and then they have reinforced the whole arrangement using a fibreglass filler. Additionally, and for the sake of comparison, they have attached four FBGs and four strain gauges to the specimen. They have distributed these sensors according to Figure 2.

**Figure 3:**
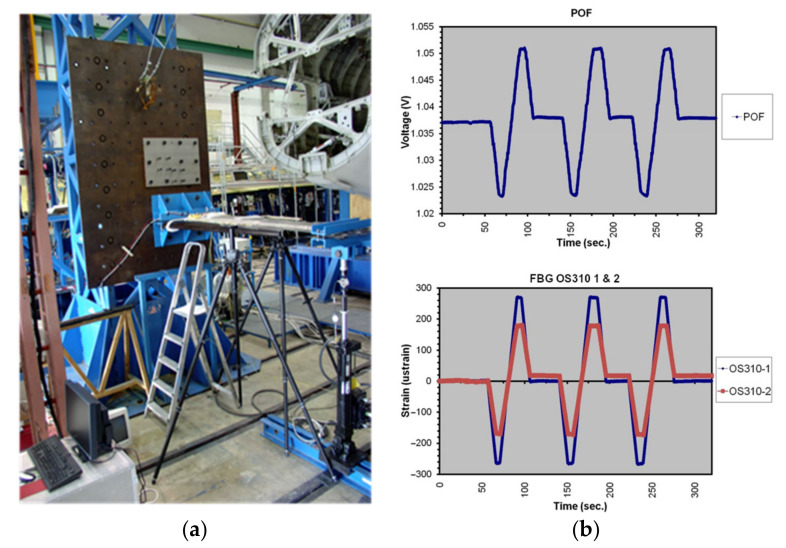
(**a**) The testing assembly. (**b**) Cyclical movements from zero to +10 cm, then to −10 cm, return to zero and so on (speed: 10 mm/s). Top: POF sensor. Bottom: FBGs 1 and 2.

Figure 3a shows the testing assembly for the aircraft flap. It is based on a framework with an interface to fix the specimen. It has a hydraulic actuator to bend the specimen (up and down). A 3D camera has also been used to control the bending of the specimen. Several tests have been made at different speeds and durations. They include single movements, cyclical movements, and systematic movements. Figure 3b also shows the results from a cyclical test. A good repeatability and linearity in the output of the POF sensor is observed. As to the maximum operating temperature, these sensors can work up to 95 °C, and, therefore, they can safely be used in real-time conditions.

**Figure 4:**
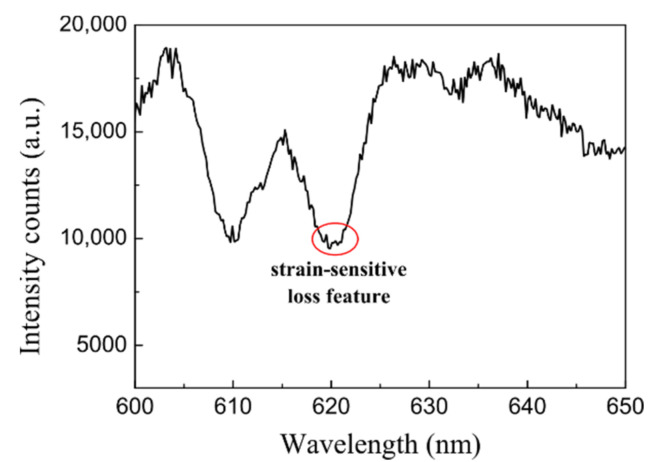
Transmission spectrum of an LPG mPOF. The loss feature wavelength corresponds to the strain-free case.

Another POF elongation sensor relies on the use of long-period gratings (LPGs) written on single-mode mPOFs [34,35]. This is possible because, as shown in Figure 4, the transmission spectra of the LPGs show loss features at the resonant wavelength (where the core mode couples to a cladding mode) and this resonant wavelength changes with the applied strain. Thus, any increase in the strain applied to the LPG implies a decrease in the resonant wavelength. In contrast, in the case of FBGs, the amount of Bragg wavelength shift with axial strain is positive.

**Figure 5:**
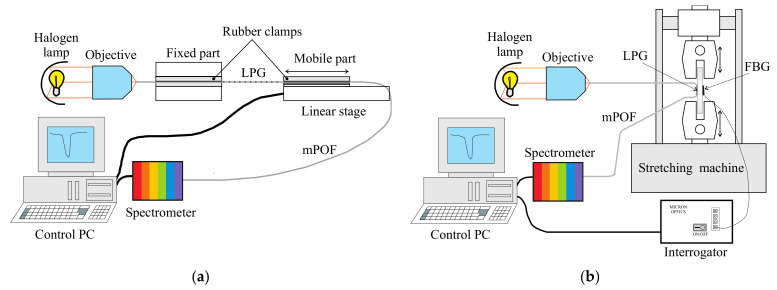
Experimental testing scenarios: (**a**) optomechanical response of the LPG mPOF between two rubber clamps; (**b**) quasi-static loading of the proof specimen.

To test the response of the sensor, two different scenarios were considered (see Figure 5). In the first one, two rubber clamps hold the LPG mPOF. The linear stage applies a uniform strain to one end in order to test the linearity of the response of the sensor (see Figure 5a). In the second scenario, the LPG mPOF is bonded to the surface of a rectangular steel plate. A silica-based FBG is also attached to the opposite surface on the steel specimen as a reference. The plate is then subjected to different loading conditions by means of a traction/compression machine (see Figure 5b).

**Figure 6:**
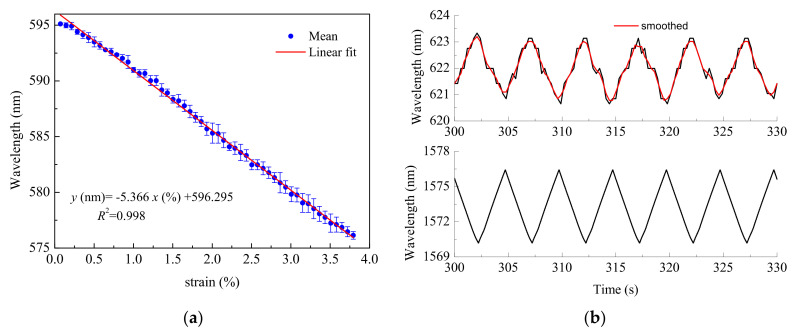
Experimental testing scenarios: (**a**) ramp-like movements; (**b**) quasi-static triangular-like loading. Maximum strain of 0.2% applied to the plate. Top: LPG mPOF. Bottom: FBG.

Figure 6a illustrates the results obtained in the first scenario for ramp-like movements under usual component testing conditions (strain values under 5%). The sensor exhibits a high degree of strain linearity. However, for small strain values, there is a non-linear behaviour attributed to the absorption of part of the stress by the rubber clamps. Figure 6b shows the results obtained in the second scenario for a triangular-like tension cycling. The upper curve corresponds to the response of the LPG mPOF sensor and the lower curve to the response of the FBG reference sensor. The signals provided by both sensors are out of phase 180° due to the opposite polarity of the slope of the wavelength with the applied strain between the LPG mPOF and the FBG. The response to the strain is linear, even though the fluctuations observed are due to the limited resolution of the spectrometer and the data acquisition sampling frequency is different to the traction frequency. Anyway, it is still possible to conclude that the LPG mPOF sensor shows an excellent behaviour for monitoring the strain.

## 3. Turbine Engine Measurements

This section describes the measurement of critical parameters to assess the turbine operation using two different sensing techniques. First, Blade Tip Timing (BTT) and Tip Clearance (TC) are obtained using a reflective intensity modulated optical fibre sensor based on a trifurcated optical fibre bundle. In the second part, a speckle−based sensing technique implemented with a dual-wavelength approach that is able to detect micro-vibrations with the accuracy and resolution required for on-board aircraft monitoring is described.

### 3.1. Measurement of Blade Parameters in Turbines

BTT and TC measurements are critical to assess the operation of turbines. Whereas the BTT measurement provides useful information for SHM, the TC measurement relates to engine efficiency. On the one hand, most BTT solutions used by industry employ several sensors mounted circumferentially and they are still based on algorithms established at the end of the 20th century, even though several alternatives are currently being explored (more details can be found in [36]). On the other hand, there are several TC measurement systems that use different technologies, such as capacitive, eddy current, pneumatic, strain gauges, electromechanical, microwave, and optical sensors (see the introduction in [37] for additional details). Nevertheless, optical sensors provide fast response, high resolution, and accuracy, and thus they are able to provide more detailed information than other commercial systems [37].

**Figure 7:**
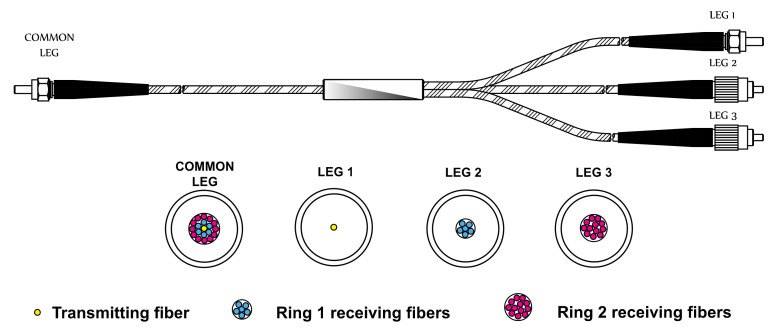
Trifurcated optical fibre bundle for BTT and TC measurements.

**Figure 8:**
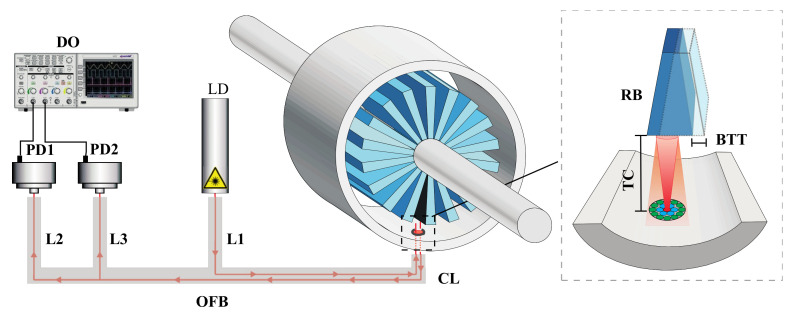
TC and BTT measurements in turbine rigs: testing assembly.

García et al. show in [38] a reflective intensity modulated optical fibre sensor. It consists of a trifurcated optical fibre bundle as presented in Figure 7. It is 3 m long and it can be made of multimode POF or GOF. The central core of the common leg corresponds to the transmitting fibre and it is connected to Leg 1. In contrast, the inner and outer rings of the fibres of the common leg collect the reflected light, and they are connected to Legs 2 and 3, respectively. Figure 8 shows the experimental set-up. It is important to note that the maximum operating temperature is currently limited to 60 °C due to the glue used to assemble the POFs in a bundle. However, since the sensors are fixed to the casing of an aircraft engine in the first stage of the compressor, temperatures will not be an issue in the following measurements. All tests have been performed in a turbine rig with a rotor of 146 blades. These blades reflect the light impinging from the illuminating fibre. The reflected light is collected by both rings of the receiving fibres. Afterwards, the photodetectors connected to Legs 2 and 3 carry out the photoelectric conversion. Finally, the digital sampling oscilloscope acquires these signals [38,39,40,41].

The BTT is associated with the arrival time of each blade. As expected, there will be differences in the BTT when changing from normal conditions to vibration conditions. Since these time differences will depend on the vibration frequency and amplitude, they can be used to predict the tip deflections of the blades. This parameter is crucial, because blade vibrations may induce cracks in the blade that would finally lead to the engine failure.

**Figure 9:**
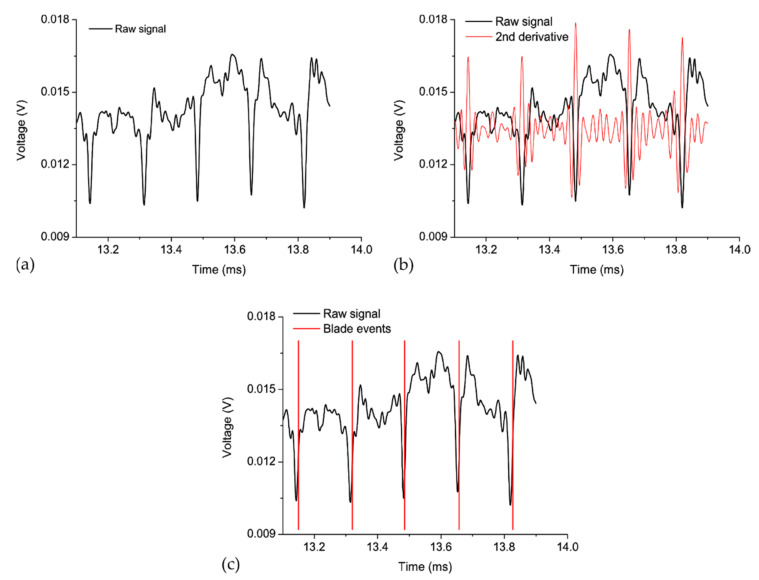
Data processing in order to estimate the BTT: (**a**) raw signal of the quotient from both photodetectors; (**b**) second derivative of the raw signal; (**c**) blade events calculated form the second derivative.

The authors of the study have estimated the tip deflection of each blade by the following procedure (see Figure 9): first, they compute the quotient of the voltages from the photodetectors connected to Legs 2 and 3 (for the reasons explained below). The obtained result is the raw signal depicted in Figure 9a. Next, they calculate the second derivative of this raw signal, as shown in Figure 9b. Then, they obtain the blade arrival event by choosing a threshold value for the second derivative (see Figure 9c). Finally, they multiply the arrival times by the rotation speed. The final result is the deflection or deviation of each blade from its theoretical equilibrium position. In addition, a fast Fourier transform of these deflection values gives the travelling wave spectrum. This spectrum is the average tip amplitude of all the blades, and it is useful for early detection of cracks and engine failure.

The TC is the distance between the casing of the engine and the tip of the blade. Since the distance measured by a reflective intensity modulated optical sensor depends on the light intensity received by the sensor, any undesired power change in the light-emitting source would lead to an incorrect measurement of the distance to the target. (Notice that there are additional sources of light fluctuations that depend on the target surface reflectivity, on the optical fibre loss, or on the misalignment between the probe surface and the target surface.) The authors avoid this problem with the use of the trifurcated fibre bundle: they divide the signals converted by the photodetectors connected to the outer and the inner receiving fibres, cancelling the effect of any undesired change in the light; therefore, the quotient will be directly related to the distance to the target [37,42,43].

**Figure 10:**
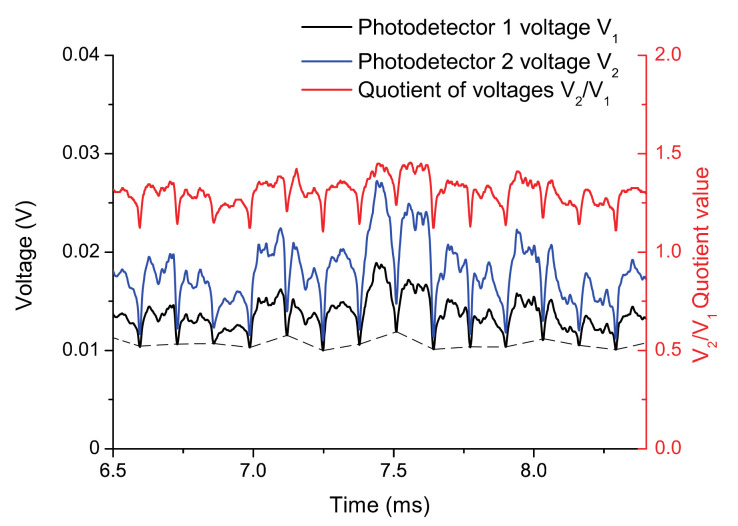
TC obtained as a function of the quotient of two photodetector voltages (V_2_/V_1_).

Figure 10 shows the raw signal of 15 consecutive blades obtained from both photodetectors as well as the quotient (in red) when the turbine rotates at 3148 rpm. The TC is obtained from the quotient of the photodetector voltages, according to a calibration curve measured previously [38]. The response of the optical sensor was compared to that of a discharging probe sensor, which is a device typically used for TC measurements. The differences between them are in the order of some tens of microns and the relative difference is less than 3% although the optical fibre sensor gives accurate information of every blade.

### 3.2. Micro-Vibration Sensors

When a highly coherent light propagates through an MMF via a number of modes, interference between these modes yields a speckle pattern, a so-called modal noise, at the fibre end-face, firstly reported by [44]. This speckle pattern is extremely sensitive to the phase difference among the propagated modes, which can arise from time-varying fibre disturbances resulting from external forces or thermal drifts, among others. Although seeing this effect as a drawback for high-speed optical communication systems through MMFs, immediately the optical sensing community envisioned this physical effect for boosting a new research field to develop fibre-optic sensor potentially suitable to measure a wide range of measurands (pressure, temperature, vibration, etc.). However, due to the high sensitivity of the speckle pattern changes, speckle-based sensing can be a drawback in final applications due to the random nature of the interference of the fibre modes produced by ambient perturbations as well as the modal noise of the light source.

Speckle-based POF sensors have been developed to monitor pressure in petrol pipes [45], to measure the temperature at the surface of a metal plate [46], or to quantify strain [47]. For instance, researchers have reported on speckle analysis to monitor the vital signs of patients [48,49] and its use in combination with FBGs to measure strain [50]. EHM in aircrafts requires technologies that provide early warning of faults and the capability to monitor the development of faults so that timely action can be taken. Problems such as dirt, fouling, erosion, oxidation, corrosion, material stress, foreign object damage, domestic object damage, worn bearings, worn seals, excessive blade TCs, burned or warped turbine vanes or blades, partially or wholly missing blades or vanes, plugged fuel nozzles, cracked and warped combustors, and cracked rotor discs or blades could cause failures in engines. Mastering accurate microvibrations on-board future airborne systems [51] is still a key issue for future aircrafts, requiring the ultimate accuracy for early fault detection. Most solutions reported are based on BTT measurement [52,53] as well as piezoelectric accelerometers located on the front and rear part of the engine [54], although both suffering from the small vibration magnitudes, thus hampering an accurate vibration analysis. Novel Plastic Optical Fibre FBGs (POFBGs) are also proposed, capable of measuring vibration amplitudes ranging from 1 g to 15 g with a linear response [55], although they require a complex and costly reception stage. Estimations of the instantaneous angular speed through the aircraft engine vibration signal is also a challenge [56]. First-order critical engine vibration frequencies of interest range from units of hertz up to around few hundreds of hertz [57]. SHM has also been proved for airborne components using speckle and POFs [58] with maximum frequency vibrations of 15 Hz, limited by the frame rate of 30 Hz of the camera employed. All of them operate analysing only single wavelength speckle patterns, thus being the detected measurand resolution’s limit.

**Figure 11:**
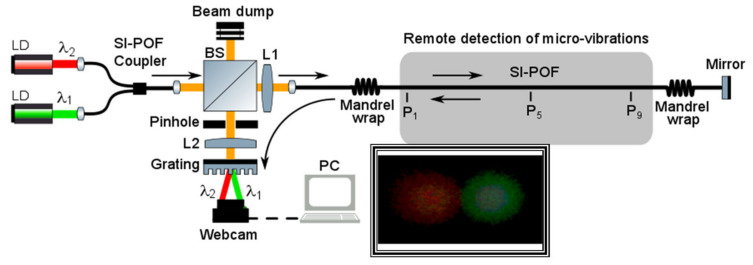
SI−POF-based micro-vibration sensor. L1 and L2: optical lenses. Inset: Example of the speckle patterns of λ_1_ (right, 520 nm) and λ_2_ (left, 650 nm) recorded by the webcam. P1, P5, and P9: spatial points where piezoelectric stacks induce periodic vibrations.

Recent advances in speckle sensing propose a dual-wavelength speckle-based sensing technique to improve the robustness to modal noise, thus allowing to increase the system accuracy as well as enhancing the system resolution to be suitable to fulfil on-board aircraft micro-vibration monitoring requirements. A scheme of the setup implemented is shown in Figure 11, with application for remote detection of periodical micro-vibrations [59]. A compact reflective topology [60] is designed, thus performing a centralized interrogation unit for multiple micro-vibration detection with enhanced sensitivity. The reflective scheme also opens up the possibility to locate the relative position of the perturbation source by analysing the speckle pattern variance of both wavelengths [61], a feature restricted in single-wavelength speckle-based approaches.

Two laser diodes (LDs), with λ_1_ = 532 nm (Green) and λ_2_ = 650 nm (Red), respectively, were multiplexed using a 50:50 coupler. The light beam composed by λ_1_ and λ_2_ was next directed to a 3-dB loss beam splitter cube BS, so that 50% of the light’s intensity was injected into the sensing fibre lead. A standard PMMA SI−POF fibre with 980/1000 μm of core and cladding diameter, respectively, also including a jacket with 2.2 mm of diameter was used as the sensing fibre lead. The fibre deployed had a total length of 11 m and included two mandrel wraps to improve the modal distribution along the fibre and to eliminate possible non-guided modes produced by the external disturbances along the fibre. Finally, the mirror at the farthest end-face of the SI−POF reflected the light back to the fibre lead, reaching the BS cube located at the interrogation unit that deflects 50% of the reflected intensity to the webcam. The light beam reaching the webcam contained the speckle patterns of both λ_1_ and λ_2_ (please refer to the inset of Figure 11). Both patterns were then demultiplexed and focalized on different spatial locations of the same webcam by using a transmission-customized diffraction grating with a groove density of 1200 lines/mm [61]. Therefore, the proposed system allows analysing two independent speckle patterns recorded at the same instant. This is a very useful performance to minimize the great impact of the random interference of fibre modes, i.e., modal noise.

Periodic vibrations are generated using a piezoelectric stack at three points (named P_1_, P_5_, and P_9_, respectively) along the SI-POF fibre lead for testing purposes, separated in steps of 4 m each. The piezoelectric stack is excited with a sinusoidal signal, thus generating a spatial displacement normal to the fibre longitudinal axis. Frequency vibrations under testing were limited to the camera speed. The proposed sensor measures absolute spatial displacements with respect to the initial spatial position.

**Figure 12:**
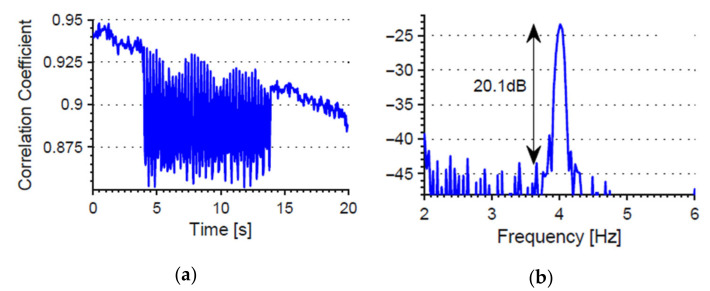
(**a**) Temporal behaviour of the correlation signal (mixed signal); (**b**) power spectral density. The vibration source is located at point P_5_.

The proposed method for remote detection of micro-vibrations computes first the correlation factor between the time-varying speckle patterns recorded by the webcam at the two wavelengths related to a reference pattern, then it takes the average of 20 consecutive speckle patterns, and afterwards it computes the resulting mixed correlation signal of the latter. Figure 12 shows the results of this mixed correlation signal after data processing. The experiments show a 2.6 dB increase in the signal-to-noise ratio compared to the results obtained from the correlation signal obtained from the single-wavelength approach. Micro-vibration resolutions below 0.2 μm were achieved, well below the measured and estimated displacements in the aircraft’s structural bodies of interest [62,63,64], thus making this method suitable for aircraft vibration monitoring applications. There is no need for a bare fibre or reducing the number of modes to achieve an enhanced resolution.

## 4. Level Measurement in Tanks

**Figure 13:**
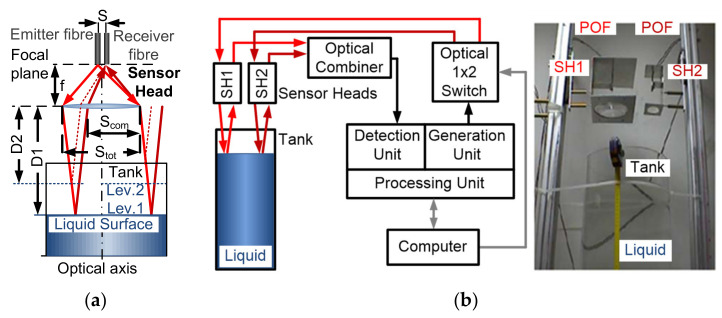
Liquid-level sensor: (**a**) Sensor heads: incident (−−−) and reflected (-·-·-) beam path depending on the location of the lens and the emitter and receiver fibres. (**b**) System architecture and picture of the two sensor heads at the top of the tank.

In aircrafts, the main disadvantage of electronic sensing solutions is the risk of explosion as the electrical current must be introduced into the flammable (or simply conducting) liquid. Optical fibres have been used for measuring liquid levels in many forms: some are non-intrusive sensors based on light attenuation while passing tank walls, as in [65], but are only useful in transparent tanks; other non-intrusive, inherently safe POF-based liquid-level sensors use POF fibres for greater light reception [66]. Its principle of operation is based on the attenuation suffered by the light transmitted from the emitter fibre to the received fibre when it is reflected by the liquid surface. The two fibres are placed in the focal plane near the lens focus; in this way, the reflected beam suffers a lateral displacement that depends on the distance between the lens and the liquid surface, as is shown in Figure 13a. The lens determines the range and sensitivity of each sensor. In addition, the system includes a 1 × 2 liquid crystal-based optical switch that allows performing a time division multiplexing technique between the two sensor heads (see Figure 13b), although easily scalable [67]. A resolution of around 1 cm of liquid height is achieved. The liquid level varied from 1.90 m to 1.46 m in height. However, random vibrations, such as in transportation systems, can introduce additional errors.

Intrusive schemes to increase the sensitivity, bends [68], plus the cladding removal and partially polished core [69], can be easily done on POF fibres. An application for fuel-level measurements in paramotoring and powered paragliding is reported in [70], where the stripped POF liquid level gauges with different shapes are proposed with sensitivities at reception of 0.5 V per bend without the need of partially removing the fibre core.

**Figure 14:**
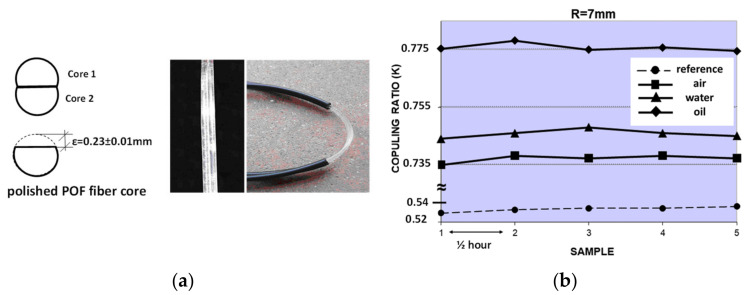
(**a**) Scheme of the proposed self-referenced liquid-level sensor and picture of the customized POF coupler-based sensor. (**b**) Measurements of the sensor coupling ratio for different surrounding specimens (air, water, and oil). The bending radius was 7 mm. The time between samples was ½ hour.

When a higher precision is required, self-reference techniques should be considered [71,72], especially for intensity-based optical sensors, where an additional fibre lead or optical path is usually needed to assure the self-reference property, i.e., to normalize out any laser output fluctuations that would otherwise be interpreted as variations in the measured magnitude. A liquid-level sensor based on the changes of the coupling ratio (K) of a customized POF coupler is presented in [73]. The coupling ratio (K) acts as the self-referenced sensitive parameter itself, thus showing no dependency of the injected optical power fluctuations or random power losses in the sensor network. The sensor is made of standard SI-POF plus cladding removal and a partially polished core to increase the sensitivity with respect to the measurand, i.e., the surrounding liquid. Both POF coupler arms have been joined by gluing over the length of the sensing area with a no-gap interface between the two fibre cores. Schematics of the POF sensor as well as a picture of the real device manufactured are depicted in Figure 14a.

Experimental measurements to show the proof-of-concept of the proposed sensor were carried out. The coupling ratio values for *Carthamus tinctorius* oil (*n* = 1.466) and water besides the air surrounding the sensor for different bending radii (*R*) (see Figure 14b for the *R* = 7 mm case) were tested. The time between samples was ½ hour. The reference measurement refers to an unbent POF sensor with no surrounding liquid, i.e., air. The self-reference property of the proposed liquid level was tested for induced optical link losses up to 4 dB.

## 5. Temperature Measurement in Aircraft Structures

Temperature is one of the parameters to be controlled in different applications, such as in the automotive industry, air-conditioning control, chemical processes, food storage, or medical applications, among others [74]. Traditional temperature sensors, such as resistance temperature detectors, thermistors, or thermocouplers, are not suitable when they are used in harsh environments or in the presence of electromagnetic disturbances. This problem can be solved by using FOSs because they use optical signals instead of electrical ones, and thus have intrinsic safety and electromagnetic immunity. Different types of FOSs are proposed for measuring the temperature, such as interferometric configurations, like Mach–Zehnder [75], Fabry–Perot [76], or Michelson [77], or intensity FOSs as those based on light attenuation [78], frustration of total internal reflections [74,79], control mode coupling [73], heat radiation [80,81], FBGs [82], fluorescence [83], or non-linear effects [84], among others. In addition to this, optical fibre pyrometers that measure the energy emitted by bodies at different temperatures are used for non-contact temperature measuring [85]. Despite interferometric sensors having a high sensitivity, they require complex interrogation equipment. On the other hand, intensity-based sensors allow to obtain the measurement with simpler signal processing and easy implementation. However, the main drawback of the intensity-based sensors is the need for a self-referencing scheme [86,87] in order to avoid undesirable perturbations in the optical power loss that can distort the measurements.

**Figure 15:**
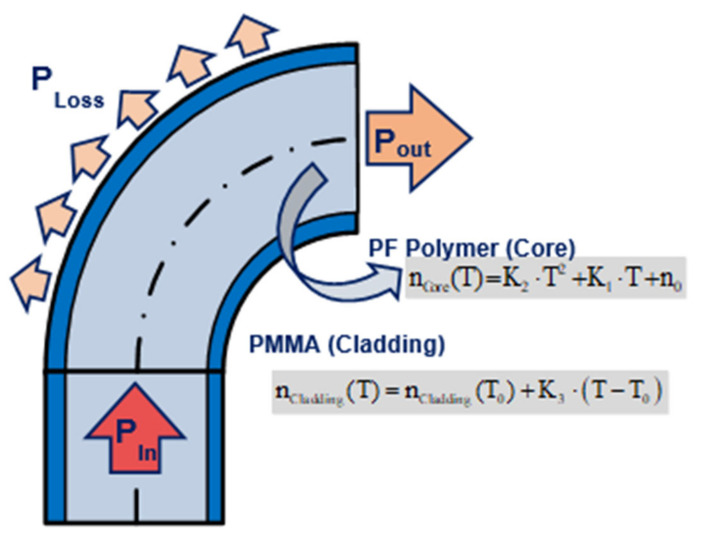
A macro-bending temperature sensor, and its principle of operation.

A temperature sensor that consists of a macrobend made with POF is presented in [74]. Its operation principle is based on bending the MMF, as it is show in Figure 15. The local numerical aperture (NA) on the curvature changes with the temperature due to the thermo-optic coefficients of the materials. The thermo-optic effect of POF is larger than in glass fibre in more than one order of magnitude, thus its refractive index is more sensitive to temperature changes (the reader is referred to the thorough review by Peters in [27] for further details). In addition, POF is less fragile and easier to handle, both good properties for developing sensors. The macrobend sensor is manufactured with a 180° loop.

**Figure 16:**
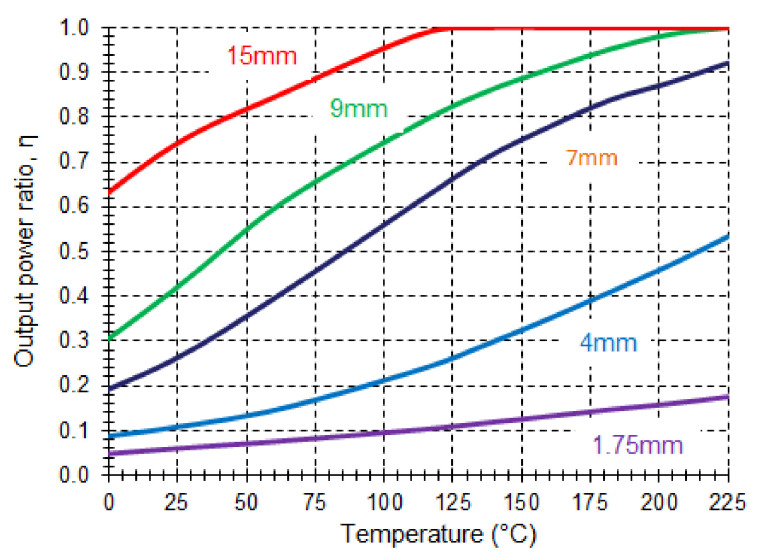
Macro-bending temperature sensor: ratio of output power to input power versus temperature.

The ratio of output power to input power changes with temperature. This variation depends on the radius of the curvature, as is observed in Figure 16. There is a trade-off between sensitivity, linearity, and total losses that needs to be considered when designing the sensor. A bend radius of 2 mm provides a good linearity. Different self-reference techniques have been proposed, such as using a dummy sensor or using two wavelength bands from a sliced broadband source [88]. The obtained sensitivity is 1.92 × 10^−3^ °C^−1^ when the dummy sensor is used and 8.95 × 10^−4^ °C^−1^ in the two wavelengths set-up.

More recently, another temperature sensor based on the variation in the POF’s mechanical properties with temperature has been presented in [89]. When a constant stress is applied to the fibre, the fibre output optical power changes due to the stress-optical effect. The stress is also obtained by means of a 180° curvature; the fibre is fixed for avoiding curvature variation and a lateral section in the inner part of the POF fibre is used for creating a sensitivity zone. The sensor can operate at temperatures up to 110 °C; the measured sensitivity is 1.04 × 10^−3^ °C^−1^ and a linearity of 0.994.

**Figure 17:**
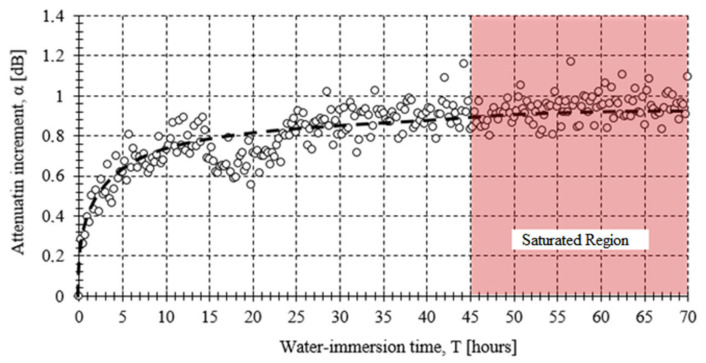
Attenuation variation versus immersion time with water at 50 °C.

A consideration to take into account when using POF sensors into aqueous media is that the water absorption can greatly affect the sensor performance during the first immersion hours. Test of a macrobend sensor dipped in water is reported in [90]. We have developed different measurements with a macrobend sensor dipped in water at 50 °C and using an oven to keep the temperature stable. The larger variation in the water absortion occurs in the first 20 h of immersion and the stabilization arrives after 45 h, as is shown in Figure 17. The absorption saturation level is close to 1.35%, a value reached when there are no longer mass changes observed in the sample. After that time, there are attenuation variations of 0.06 dB. They are equivalent to those presented in dry sensors when an increment of air temperature of around 4 °C happened.

On the other hand, if high tensile stress is applied to the POF sensor, small temperature increments can appear. The characterization of different SI-POF samples [91] shows temperature variations <0.5 °C in the elastic regime and variations >3 °C in the plastic regime [92].

## 6. Auxiliary Technologies

This section describes auxiliary technologies that allow remote feeding and interrogation of sensor networks through POFs. First, the methodology to enable POF links for power distribution through a power-supply method is presented. Second, the impact of air-gap connectors in a POF-based data network is statistically analysed using a simulation methodology to engineer a typical avionics system.

### 6.1. PoF Using POF for Avionic Sensors

Optical-fibre-linked power-by-light systems, i.e., PoF systems, usually consist of a high-power laser diode (a monochromatic source), a transmission line (an optical fibre), and a converter (i.e., a photovoltaic cell). The transmission of electrical power over optical fibres is already a well-established technology. Many commercial systems are based on the combination of low-loss silica glass fibres and high-power infrared laser diodes. They can convey up to 1 W of power over a minimum distance of 1 km [93,94,95,96], even though their system energy efficiency (SEE) is approximately 1% (SEE is the ratio between the energy delivered to the remote node and the energy provided by the high-power source at the transmitter). Nevertheless, new optimized PoF designs can increase this figure of merit to one order of magnitude [97]. There is a growing interest in optically powered systems motivated by recent advances in power-efficient innovative hardware, low-energy medium access control protocols, and power consumption savings, both in electronics and in the remote nodes of the Internet-of-Things (IoT). In that way, POF-based power-delivery systems are excellent candidates to boost short-range distance applications in aircrafts, as well as in home-networking fields in combination with IoT remote nodes. However, in comparison to their glass counterparts, PoF systems over POF are limited by the maximum operating temperature of the polymer, which is closely related to its low glass transition temperature. As a consequence, this maximum operating temperature imposes several restrictions to the maximum optical power density of the polymer before fibre damage [98]. Furthermore, there are additional penalties in the power delivery efficiency due to the greater attenuation coefficient of the POF.

López-Cardona et al. have reported in [99] electrical powers of around 50 mW over 50-m-long 120 μm core diameter PF GI-POFs with a high-power laser operating at 808 nm and an input optical power of 197 mW. The photovoltaic efficiency is around 38%. The photovoltaic efficiencies at 808 nm are higher than at 1300 nm, driving the use of PoF signals at 808 nm over PF GI-POFs and maximizing the system energy efficiency [100]. The experiments also estimate a maximum optical power of 528 mW before PF GI-POF fibre damage, being critical in GI profile optical fibres due to the effective mode field diameter. Thus, large core diameter SI-POFs are able to maximize the remote power delivery via PoF, and some proof-of-concept tests have been carried out.

**Figure 18:**
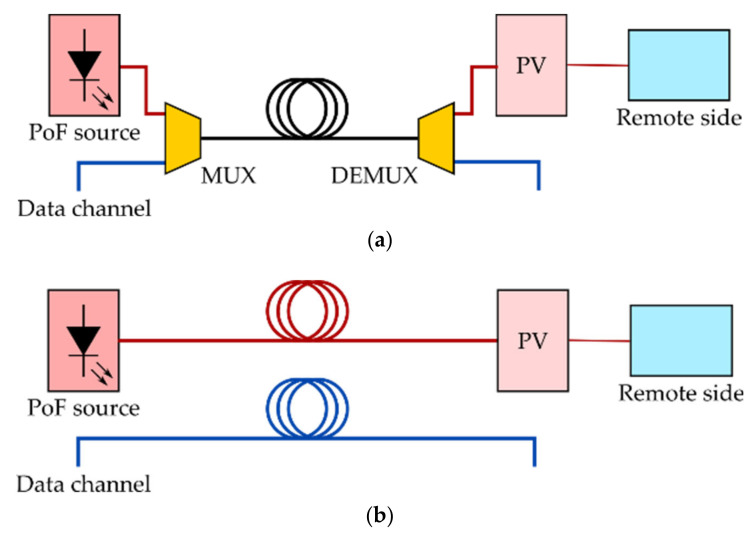
Different powering schemes: (**a**) shared scenario; (**b**) dedicated scenario. PV: Photovoltaic cell.

For many years, large-core SI-POFs have been considered a potential candidate to deploy a dedicated backbone, guaranteeing gigabit speeds in many applications such as home networking [101] and automotive [14] fields thanks to their easy installation and high bending tolerance. Coexistence of PoF signals and data traffic over the same POF fibre lead is then envisaged, with the need of MUX/DEMUX devices to combine/split the data traffic as well as the PoF signal into the same POF fibre (shared scenario); in contrast, in a dedicated fibre scenario, two independent fibres would be used for data transmission and PoF delivery [102]. Both scenarios are shown in Figure 18.

MUX/DEMUX devices in such types of systems are critical as they should have acceptable insertion losses (IL), high crosstalk (CT), and, in addition, be able to handle high PoF power levels. PoF might have a direct impact on the data signal quality, and, therefore, additional experiments have been reported in [103] in order to test the potential of Gbit/s transmission over 1-mm-diameter SI-POFs in the presence of optical power delivery signals.

Fahad Al-Zubaidi et al. proposed and analysed in [102] a real-time WDM system with a maximum capacity of 5 Gb/s over a 10-m-long SI-POF in combination with PoF capabilities. An example of a shared scenario includes data at 650 nm and a PoF signal generated by an LD emitting at 405 nm. Data and power channels are multiplexed and launched into a 10-m-long PMMA SI-POF link through a MUX device. This MUX device is based on reflective diffraction grating technology, and it can accommodate up to six channels with a low IL of approximately 4 dB [104]. The DEMUX device is implemented using the same technology. BER measurements for different PoF levels show no impact of PoF on the data signal quality transmitted at 650 nm. In all cases, BER is lower than 1 × 10^−10^, making possible a free error transmission condition with a simultaneous delivery of several mW of optical power at the fibre end. The SEE ranges from 5 to 25%. Experimental remote electrical power delivery over a 10-metre-long SI-POF has also been provided. An output power of 20 mW for the LD should be enough to fit with the experimental values. Better figures of merit could be obtained by reducing IL in the MUX/DEMUX devices: for instance, by considering the fibre bundle approach for POF multiplexing, as reported in [105], or by increasing the number of wavelengths for PoF purposes—650 nm would be kept for standard data transmission. Further details of this scalability analysis have been reported on [102].

### 6.2. Impact of Air-Gap Connectors in Aircraft Networking

Large-core PMMA POFs have been proposed as a competitive media for avionics data networks because their bandwidth-length products are sufficient for these networks and polymer fibres are flexible, resistant, and can be coupled to cost-effective devices [22]. The stringent requirements of aircraft systems, however, preclude the direct application of technology developed for other applications. In particular, environmental requirements for safety critical data communication for commercial aircrafts established a maximum temperature variation of +/−10 °C/min within a temperature range from −55 °C to 85 °C; that is, just inside the operation temperature limits for standard PMMA POFs. Heat-resistant PMMA fibres with thermoset jackets have a higher temperature limit of 105 °C, as thermoset polymers crosslink together during the curing process to form an irreversible chemical bond that eliminates the risk of melting when heat is applied [106]. Although their attenuation and bandwidth are slightly poorer than those of standard fibres, it was shown that they are able to withstand temperatures up to 125 °C, beyond their nominal limit, with negligible changes in performance [107,108].

Apart from the harsh environmental requirements, another distinctive aspect of avionics data networks is that the design of their physical layer is based on multiple fibre segments joined by connectors. Thus, an ample power budget is needed to cope with fibre lengths up to 100 m and many in-line connectors. Experimental measurements of POF connector losses in normal conditions have been obtained for different types of connectors and fibre termination procedures and found to be rarely below 1 dB [109]. In aircrafts, the presence of air-gaps in the interconnect ferrules (in order to prevent end-fibre damage by friction) is combined with vibration-induced misalignments to further increase connector insertion loss. Cherian et al. tested the endurance to temperature and vibrations of a polymer fibre optic harness, including different connector types. Following the methods defined in the aircraft military standards (MIL-STD-1678), they found a stable behaviour of connectors to temperatures up to 85 °C, but they measured insertion loss changes from 0.5 to 1 dB, depending on connector type [27]. Apart from the increase in optical power loss, experimental results confirm that connectors also change the system bandwidth depending on their positions in a POF link [110,111].

In this context, a simulation framework that describes the light propagation in POFs and accounts for the impact of the system components on the link performance, in order to test the design reliability before its implementation, is indispensable. We have addressed the need for this framework adapting a previously developed matrix model for large-core PMMA fibres and components to system engineering of avionics SI-POF links [112]. In this sub-section, we briefly describe how this model has been applied to evaluate the performance of a typical avionics system with several POF segments and including a number of connectors. Then, our latest results on the impact of air-gap value on link performance are shown in more detail.

In our simulation framework, the effects of light propagation in POFs are modelled using a matrix approach that renders a flexible implementation of the diffusion equation by means of the fibre characteristic matrix obtained from experimental measurements [113]. This method is also capable of accommodating the effects of other system linear components, such as connectors, that are also modelled by matrices [114]. According to the results obtained by integrating the matrix framework into commercial software, SI-POF links may support significantly higher bit rates than the 4 Mb/s rate needed for basic data and airplane health monitoring applications, provided that a sufficiently high power is launched into the fibre.

Mateo et al. propose in [115] an upgrade of the connector matrix model that incorporates lateral and longitudinal misalignment losses based on the calculation of the radiated angular intensity distribution using the Hankel transform. This feature allows modelling air-gap connectors and statistically variable positional shifts to obtain realistic simulation results of the performance of POF avionics links. In a recent work, the impact of this variability in transmission properties has been assessed using the propagation matrix framework combined with the upgraded ST connector model to simplify and speed up the Monte Carlo simulations [116]. The longitudinal offset (*z*) is drawn from a Gaussian distribution with a mean of 150 μm (a value typical for an air-gap connector) and a standard deviation of 31 μm. Lateral misalignments (*x* and *y*) have also Gaussian distributions with the same standard deviation, but a zero mean, which lead to a Rayleigh-distributed radial offset. These values are chosen to comply with the tolerances defined in the IEC-60793-2-40 specifications, A4a.2 fibre class.

López et al. have analysed in [116] a typical avionics SI-POF link of 42 m in various segments joined by connectors. The angular intensity distribution launched into the fibre was modelled using measurements obtained for the transmitter of a commercial transceiver based on a VCSEL emitting at 645 nm [117]. The receiver was modelled as a wide area detector whose driver electronics circuit poses no extra limitation to the fibre frequency response as, for these lengths, fibre modal dispersion is the major contributor to frequency response degradation. Statistical distributions of power loss and bandwidth in the different scenarios were extracted from the resulting output intensity distributions and frequency responses, respectively, in order to assess the impact of the number and position of the connectors.

The results show that, as expected, increasing the number of connectors, the power loss increases and the bandwidth decreases. The standard deviations of both properties also increase with the number of connectors. It also was found that changes in the connector position do not alter the power loss, but they have a significant impact on frequency response and bandwidth—higher bandwidths are obtained if the connectors are closer to the detector. Some of these issues would be solved using methods for low loss splicing of POF segments. Although there are some proposals of cost-effective methods to join to PMMA fibre segments [118], they are not yet able to withstand the rough avionics environments, such as those designed for glass fibres [119].

For this reason, and since the drastic increase in power loss with the number of connectors is related to the relatively large air-gap of 150 µm, the authors have analysed the effect of changing the longitudinal fibre-to-fibre offset over power loss and bandwidth in order to assess the impact of the air-gap in transmission properties. The simulated link has a total length of 42 m with six 7-m fibre segments joined by 5 connectors. Now, in addition to the typical 150 µm longitudinal offset, we considered another two values for the air-gaps: 200 and 100 µm. These three values are introduced as the means of the Gaussians distributions with standard deviations of 31 µm. Longitudinal fibre offsets are drawn from these three distributions to obtain the separate statistical distributions of power loss and bandwidth for the three different values of air-gap. The other conditions, including lateral *x* and *y* misalignments, are the same used in our previous study and are described above.

**Figure 19:**
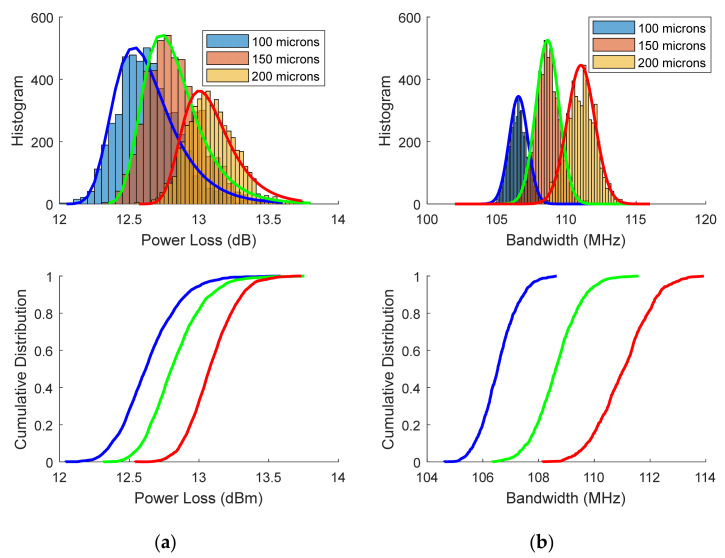
Histograms (**top**) and cumulative distributions (**bottom**) obtained for three different air-gaps (100, 150, and 200 microns—blue, green, and red curves, respectively): (**a**) power loss; (**b**) bandwidth, for a link of six 7-m segments joined by 5 air-gap connectors.

The top of Figure 19 shows the histograms for the power loss and bandwidth obtained for three different air-gaps (100, 150, and 200 microns). The lines of the histograms correspond to the fit of the extreme value distributions for the power loss and normal distributions for the bandwidth. The bottom of the figure shows the corresponding cumulative distributions. The rather high power loss accounts for both the 42 m fibre attenuation and the insertion losses of the 5 air-gap connectors.

**Figure 20:**
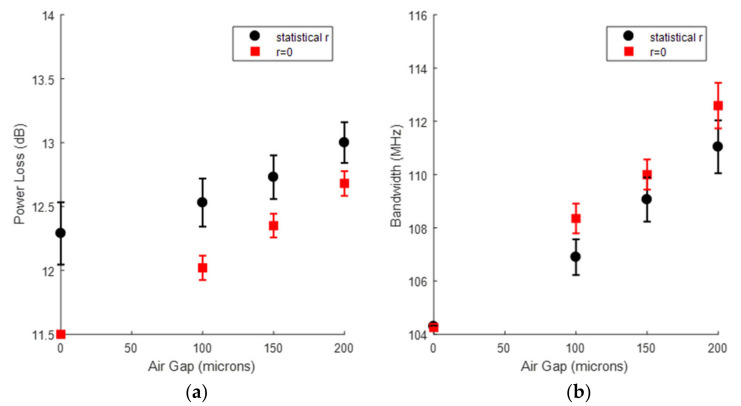
Analysis of the effects of the longitudinal gap in the presence and in the absence of radial misalignments: (**a**) power loss; (**b**) bandwidth, for a link of six 7-m segments joined by 5 air-gap connectors.

These results show that power loss and bandwidth increase with the air-gap. Both effects are related to the filtering out of higher order modes when the fibre ends are separated by the air-gap. A similar effect occurs when the output end of a large numerical aperture POF is placed at a distance of the active area of the photo-detector, and it has been reported before [113,115]. Figure 20 reports the effects of the longitudinal gap in the presence and in the absence of radial misalignments. The black circles and bars denote the average and standard deviation of the power loss and the bandwidth obtained from the previous histograms (with all possible offsets). In contrast, the red squaresdenote the power loss and the bandwidth when there is no radial offset (*r* = 0).

In the absence of an air-gap, the bandwidth is indifferent to radial misalignments. As the longitudinal gap increases, the bandwidth also increases, but it is slightly higher when there is no radial offset. As for the power loss, it is lower when there is no radial offset, but its increase with the air-gap value is steeper.

**Figure 21:**
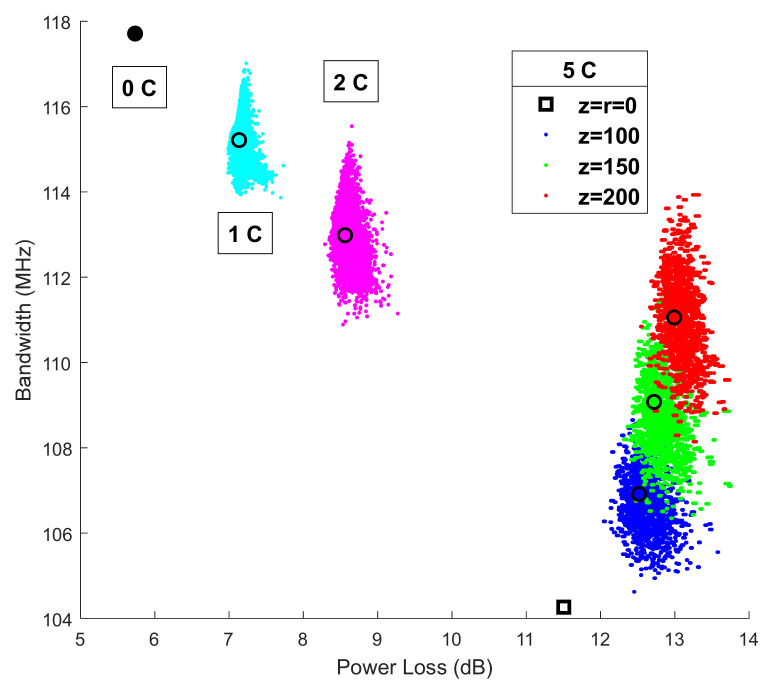
Scatterplot of the bandwidth vs. the power loss for different lay-outs: 42-m fibre with no connectors (0C), two 21-m segments joined by one connector (1C, 150 µm air-gap), three 14-m segments joined by two connectors (2C, 150 µm air-gap), and, finally, six 7-m segments joined by five connectors (5C) and three different air-gap values (100, 150, and 200 µm).

Finally, Figure 21 shows a summary of the results by plotting the bandwidth versus the power loss for the 42-m fibre in segments joined by 5 connectors (5C) with the different air-gaps considered (*z* value). The individual data are represented as points with different colours depending the air-gap value: blue for 100 µm, green for 150 µm, and red for 200 µm. The hollow square shows the deterministic result obtained for 5 connectors with no air-gap nor radial misalignment (*z* = *r* = 0). Hollow circles show the average for each condition. To complete the analysis, we have also included the results obtained in our previous study for different numbers of connectors. The case with no connectors (0C) is shown as a filled circle. The data obtained with one connector joining two 21-m segments (1C) and two connectors joining three 14-m segments (2C) are shown as cyan and magenta dots, respectively. These cases were simulated only for a *z* = 150 µm air-gap. Again, the hollow circles show the average values.

On the one hand, the connectors introduce a very high power loss, and the extra loss due to the air-gap is comparatively small. Notice that even for a five-connector link (5C) with neither air-gaps nor radial offsets (5C *z* = *r* = 0), it is 6 dB higher than for a link without connectors (0C) while there is only a 1.5 dB increase in power loss when the air-gap changes from 0 to the largest separation (5C *z* = 200). On the other hand, there is a substantial increase in the bandwidth for a five-connector link and maximum air-gap (5C *z* = 200) relative to the case with no offsets (5C); more specifically, the bandwidth for the former is of 111.5 MHz, whereas for the latter it is 104.3 MHz.

Although large-size POFs enable low-cost termination, the soft end-face of the fibre poses significant problems in the aircraft environment as interconnect ferrules using air gaps to ensure vibration stability introduce even higher power loss although accompanied by a bandwidth increase. In addition, the hostile operating environment inside the aircraft imposes constraints on the link as thermally induced stresses, such as micro bending, deformation, aging, etc., add a power penalty to the power budget. Thus, high-power sources are required, and, therefore, thermal, reliability, and eye safety issues have to be considered.

## 7. Conclusions

We have reported on the feasibility of using POF-based sensors in avionics for a large variety of applications. The use of such sensors for strain sensing in avionic structures is very promising, since the results clearly show a high sensitivity, and a high degree of signal repeatability and linearity. Fibre-based reflective optical sensors are also good candidates to monitor the TC or BTT in aero-engine applications that require noncontact measurements, small dimensions, and immunity to electromagnetic interference. In the same way, a POF can also be used effectively as a cost-effective method for sensing vibrations by detecting periodical changes in the spatial distribution of energy on the speckle pattern in the output of the fibre.

POF-based inexpensive and sensitive fibre-optic sensors have successfully been demonstrated for both temperature and liquid-level discrete-monitoring, paving the way for their use inside aircraft tanks. These sensors provide an attractive alternative to traditional measuring systems, because the medium is only exposed to the light and the plastic material of the fibre, avoiding the risk of electrical sparks that could cause a fire or a tank explosion.

We have also discussed the use of energy to feed sensor nodes remotely for in-home applications and their feasible extension to avionic environments. It is possible to integrate PoF solutions in POF communication links with no impact on the data transmission and delivering at the same time optical power levels up to several mW.

Finally, we have analysed the effects of air-gap connectors in the performance of the aircraft networking. We have demonstrated that the impact of the air-gap is relatively small, compared to the bandwidth constraints and power loss introduced by increasing the number of connectors.

## Data Availability

The data presented in this study are available on request from the corresponding author.

## References

[1] Nicolin I., Nicolin B.A. (2019). The fly-by-wire system. INCAS Bull..

[2] Collins J.H. The challenges facing U.S. navy aircraft electrical wiring systems. Proceedings of the 9th Joint FAA/DoD/NASA Aging Aircraft Conference.

[3] Garg G.C.A., Linda R.I., Chowdhury T. (2013). Evolution of aircraft control system and fly-by-light control system. Int. J. Emerg. Technol. Adv. Eng..

[4] Gao S., Dai X., Hang Y., Guo Y., Ji Q. (2018). Airborne wireless sensor networks for airplane monitoring system. Wirel. Commun. Mob. Comput..

[5] Al-Lami H., Aslam A., Quigley T., Lewis J., Mercer R., Shukla P. (2015). The Evolution of Flight Control Systems. Technology Development, System Architecture and Operation.

[6] Mazumdar S., Chen Q. Response of contaminant detection sensors and sensor systems in a commercial aircraft cabin. Proceedings of the 10th International IBSPA Conference.

[7] Gorinevsky D., Gordon G.A., Beard S., Kumar A., Chang F.K. Design of integrated SHM system for commercial aircraft applications. Proceedings of the 5th International Workshop on Structural Health Monitoring.

[8] Miller G.E. Fiber optic sensors for aircraft. Proceedings of the SPIE O-E/Fiber LASE ’88, Fiber Optic and Laser Sensors VI.

[9] Truong T.K. Commercial airplane fibre optics: Needs, opportunities, challenges. Proceedings of the 19th International Conference on Plastic Optic Fibres and Application.

[10] López-Cardona J.D., Vazquez C., Sánchez Montero D., Contreras Lallana P. (2018). Remote optical powering using fiber optics in hazardous environments. J. Light. Technol..

[11] Koike K., Koike Y. (2009). Design of Low-loss graded-index plastic optical fiber based on partially fluorinated methacrylate polymer. J. Light. Technol..

[12] Koike Y., Inoue A. (2016). High-speed graded-index plastic optical fibers and their simple interconnects for 4K/8K video transmission. J. Light. Technol..

[13] Zubia J., Arrue J. (2001). Plastic optical fibers: An introduction to their technological processes and applications. Opt. Fiber Technol..

[14] Ziemann O., Krauser J., Zamzow P.E., Daum W. (2008). POF Handbook: Optical Short Range Transmission Systems.

[15] Van Eijkelenborg M.A., Large M.C.J., Argyros A., Zagari J., Manos S., Issa N.A., Bassett I., Fleming S., McPhedran R.C., De Sterke C.M. (2001). Microstructured polymer optical fibre. Opt. Express.

[16] Large M., Poladian L., Barton G., van Eijkelenborg M.A. (2007). Microstructured Polymer Optical Fibers.

[17] Woyessa G., Fasano A., Markos C., Stefani A., Rasmussen H.K., Bang O. (2016). Zeonex microstructured polymer optical fiber: Fabrication friendly fibers for high temperature and humidity insensitive Bragg grating sensing. Opt. Mater. Express.

[18] Fasano A., Woyessa G., Stajanca P., Markos C., Stefani A., Nielsen K., Rasmussen H.K., Krebber K., Bang O. (2016). Fabrication and characterization of polycarbonate microstructured polymer optical fibers for high-temperature-resistant fiber Bragg grating strain sensors. Opt. Mater. Express.

[19] Leal A.G., Theodosiou A., Min R., Casas J., Diaz C.R., Dos Santos W.M., Pontes M.J., Siqueira A.A.G., Marques C., Kalli K. (2019). Quasi-distributed torque and displacement sensing on a series elastic actuator’s spring using FBG arrays inscribed in CYTOP fibers. IEEE Sens. J..

[20] Ciordia O., Perez R., Pardo C. Optical communications for next generation automotive networks. Proceedings of the 22nd Microoptics Conference (MOC).

[21] López A., Losada M.A., Mateo J., Strobel O. (2016). Last mile systems, in-house-networks, LAN- and MAN-applications: Polymer optical fibers, POF. Optical and Microwave Technologies for Telecommunication Networks.

[22] Truong T.K. Boeing commercial airplanes fiber optic evolution—Applications of POF in commercial aircraft. Proceedings of the POF Symposium held at Optical Fiber Communications Conference (OFC).

[23] Global Plastic Optical Fiber (POF) Market Report 2020: All-Optical Networks, Need for Higher Bandwidth, EMI Protection, Lower Cost, Lighter Weight, Ease of Use. https://www.globenewswire.com/news-release/2020/01/29/1976753/0/en/Global-Plastic-Optical-Fiber-POF-Market-Report-2020-All-Optical-Networks-Need-for-Higher-Bandwidth-EMI-Protection-Lower-Cost-Lighter-Weight-Ease-of-Use.html.

[24] Poisel H. Optical fibers for adverse environment. Proceedings of the International Conference on Plastic Optical Fibers.

[25] Mitsubishi Chemical: POF—Polymer Optical Fiber ESKA Products. https://www.pofeska.com/pofeskae/download/pdf/catalogue/Eska_grade_list.pdf.

[26] AsahiKASEI: Asahi Kasei Corporation—Products. https://www.asahi-kasei.co.jp/ake-mate/pof/en/product/data-communication.html.

[27] Cherian S., Spangenberg H., Caspary R. Investigation on harsh environmental effects on polymer optic link for aircraft systems. Proceedings of the SPIE Photonics Applications for Aviation, Aerospace, Commercial, and Harsh Environments V.

[28] Cherian S., Spangenberg H., Caspary R. Power budget and system performance analysis of the POF link for future avionic applications. Proceedings of the IEEE Avionics, Fiber-Optics and Photonics Technology Conference.

[29] Cherian S., Caspary R. Design of polymer optical fiber data link for aircraft applications using systems engineering method. Proceedings of the 2017 IEEE Avionics and Vehicle Fiber-Optics and Photonics Conference.

[30] Peters K. (2010). Polymer optical fiber sensors—A review. Smart Mater. Struct..

[31] Bilro L., Alberto N., Pinto J.L., Nogueira R. (2012). Optical sensors based on plastic fibers. Sensors.

[32] Durana G., Kirchhof M., Luber M., De Ocariz I.S., Poisel H., Zubia J., Vazquez M.C. (2009). Use of a novel fiber optical strain sensor for monitoring the vertical deflection of an aircraft flap. IEEE Sens. J..

[33] Gomez J., Zubia J., Aranguren G., Arrue J., Poisel H., Saez I. (2009). Comparing polymer optical fiber, fiber Bragg grating, and traditional strain gauge for aircraft structural health monitoring. Appl. Opt..

[34] Durana G., Gomez J., Aldabaldetreku G., Zubia J., Montero A., De Ocariz I.S. (2012). Assessment of an LPG mPOF for strain sensing. IEEE Sens. J..

[35] Durana G., Arrizabalaga O., Arrospide E., Aldabaldetreku G., Zubia J., Azkune M. (2017). Study of the influence of various stress-based mechanisms on polarization of an SM mPOF for the development of useful devices. J. Light. Technol..

[36] Przysowa R., Russhard P. (2019). Non-contact measurement of blade vibration in an axial compressor. Sensors.

[37] Fernández-Bello R., Amorebieta J., Beloki J., Aldabaldetreku G., García I., Zubia J., Durana G. (2019). Performance comparison of three fibre-based reflective optical sensors for aero engine monitorization. Sensors.

[38] García I., Beloki J., Zubia J., Aldabaldetreku G., Illarramendi M.A., Jiménez F. (2013). An optical fiber bundle sensor for tip clearance and tip timing measurements in a turbine rig. Sensors.

[39] Garcia I., Zubia J., Berganza A., Beloki J., Arrue J., Illarramendi M.A., Mateo J., Vazquez C., Vazquez M.C. (2015). Different configurations of a reflective intensity-modulated optical sensor to avoid modal noise in tip-clearance measurements. J. Light. Technol..

[40] García I., Przysowa R., Amorebieta J., Zubia J. (2016). Tip-clearance measurement in the first stage of the compressor of an aircraft engine. Sensors.

[41] Durana G., Amorebieta J., Fernandez R., Beloki J., Arrospide E., Garcia I., Zubia J. (2018). Design, fabrication and testing of a high-sensitive fibre sensor for tip clearance measurements. Sensors.

[42] García I., Zubia J., Durana G., Aldabaldetreku G., Illarramendi M.A., Villatoro J. (2015). Optical fiber sensors for aircraft structural health monitoring. Sensors.

[43] Gil-García J.M., Solís A., Aranguren G., Zubia J. (2017). An architecture for on-line measurement of the tip clearance and time of arrival of a bladed disk of an aircraft engine. Sensors.

[44] Takahara H. (1976). Visibility of speckle patterns: Effect of the optical guide length in coherent light. Appl. Opt..

[45] Sperandio V.M., Pontes M.J., Neto A.F., Webster L.G. A new optical pressure sensor interrogated by speckles pattern for oil industry. Proceedings of the 24th International Conference on Optical Fibre Sensors.

[46] Trivedi V., Mahajan S., Chhaniwal V., Zalevsky Z., Javidi B., Anand A. (2014). Optical temperature sensor using speckle field. Sens. Actuators A Phys..

[47] Fujiwara E., Da Silva L.E., Marques T.H.R., Cordeiro C.M.B. (2018). Polymer optical fiber specklegram strain sensor with extended dynamic range. Opt. Eng..

[48] Podbreznik P., Đonlagić D., Lešnik D., Cigale B., Zazula D. (2013). Cost-efficient speckle interferometry with plastic optical fiber for unobtrusive monitoring of human vital signs. J. Biomed. Opt..

[49] Robledo Á., Sánchez I., López Cardona J.D., Vázquez C. Wearable POF-based heart-rate monitor. Proceedings of the 7th European Workshop on Optical Fibre Sensors.

[50] Rohollahnejad J., Xia L., Ran Y., Cheng R. Deformation independent FBG strain sensor based on speckle pattern processing. Proceedings of the 14th International Conference on Optical Communications and Networks (ICOCN).

[51] Banks J.C., Brought M.S., Byington C.S. Vibration sensor configuration optimization for the AV-8B F402-RR-408 engine. Proceedings of the IEEE Aerospace Conference.

[52] Liu M., Gao W., Xiao X., Teng G., Ao C., Qiao B. Tip timing based non-contact vibration measurement of aero-engine turbine blades. Proceedings of the International Conference on Sensing, Measurement & Data Analytics in the Era of Artificial Intelligence (ICSMD).

[53] Rigosi G., Battiato G., Berruti T.M. (2017). Synchronous vibration parameters identification by tip timing measurements. Mech. Res. Commun..

[54] Dulík T., Pospisilik M., Opluštil V., Beneš P. Aircraft turboprop engine vibration monitoring module. Proceedings of the International Conference on Applied Electronics (AE).

[55] Stefani A., Yuan W., Andresen S., Bang O. Polymer optical fibre Bragg grating sensors: Measuring acceleration. Proceedings of the 35th Australian Conference on Optical Fibre Technology.

[56] Wang Y., Tang B., Qin Y., Huang T. (2019). Rolling bearing fault detection of civil aircraft engine based on adaptive estimation of instantaneous angular speed. IEEE Trans. Ind. Inform..

[57] Ewins D. (2010). Control of vibration and resonance in aero engines and rotating machinery—An overview. Int. J. Press. Vessel. Pip..

[58] Reis F.M., da Costa Antunes P.F., Mendes Maia N.M., Carvalho A.R., de Brito André P.S. (2017). Structural health monitoring suitable for airborne components using the speckle pattern in plastic optical fibers. IEEE Sens. J..

[59] Pinzon P.J., Montero D.S., Tapetado A., Vazquez C. (2016). Dual-wavelength speckle-based SI-POF sensor for cost-effective detection of microvibrations. IEEE J. Sel. Top. Quantum Electron..

[60] Montero D.S., Vázquez C. (2013). Remote interrogation of WDM fiber-optic intensity sensors deploying delay lines in the virtual domain. Sensors.

[61] Pinzon P.J., Garcilopez I.P., Vazquez C. (2015). Efficient multiplexer/demultiplexer for visible WDM transmission over SI-POF technology. J. Light. Technol..

[62] Corda S. (2002). In-Flight Vibration Environment of the NASA F-15B Flight Test Fixture, NASA/TM-2002-210719.

[63] Sivakumar S., Haran A.P. (2015). Aircraft random vibration analysis using active landing gears. J. Low Freq. Noise Vib. Act. Control.

[64] Draper C.S., Bentley G.P., Willis H.H. (1937). Vibration measurement in flight. SAE Trans..

[65] Singh H.K., Chakroborty S.K., Talukdar H., Singh N.M., Bezboruah T. (2010). A new non-intrusive optical technique to measure transparent liquid level and volume. IEEE Sens. J..

[66] Vázquez C., Gonzalo A., Vargas S., Montalvo J. (2004). Multi-sensor system using plastic optical fibers for intrinsically safe level measurements. Sens. Actuators A Phys..

[67] Lallana P.C., Vazquez C., Montero D.S., Heggarty K., Vinouze B. Dual 3 × 1 multiplexer for POF networks. Proceedings of the 16th International Conference on Plastic Optical Fibres (ICPOF 2007).

[68] Zhang H., Feng L., Hou Y., Su S., Liu W., Liu J., Xiong J. (2015). Optical fiber liquid level sensor based on macro-bending coupling. Opt. Fiber Technol..

[69] Lomer M., Arrue J., Jáuregui C., Aiestaran P., Zubia J., Lopez-Higuera J. (2007). Lateral polishing of bends in plastic optical fibres applied to a multipoint liquid-level measurement sensor. Sens. Actuators A Phys..

[70] Montero D.S., Lallana P.C., Vázquez C. (2012). A polymer optical fiber fuel level sensor: Application to paramotoring and powered paragliding. Sensors.

[71] Murtaza G., Senior J.M. (1994). Referenced intensity-based optical fibre sensors. Int. J. Optoelectron..

[72] Montero D., Vázquez C. (2020). Self-referenced optical networks for remote interrogation of quasi-distributed fiber-optic intensity sensors. Opt. Fiber Technol..

[73] Montero D.S., Vázquez C., Möllers I., Arrúe J., Jäger D. (2009). A self-referencing intensity based polymer optical fiber sensor for liquid detection. Sensors.

[74] Moraleda A.T., García C.V., Zaballa J.Z., Arrue J. (2013). A temperature sensor based on a polymer optical fiber macro-bend. Sensors.

[75] Wang Y., Li Y., Liao C., Wang D.N., Yang M., Lu P. (2009). High-temperature sensing using miniaturized fiber in-line Mach-Zehnder interferometer. IEEE Photonics Technol. Lett..

[76] Choi H.Y., Park K.S., Park S.J., Paek U.-C., Lee B.H., Choi E.S. (2008). Miniature fiber-optic high temperature sensor based on a hybrid structured Fabry-Perot interferometer. Opt. Lett..

[77] Corke M., Kersey A.D., Jackson D., Jones J. (1983). All-fibre ‘Michelson’ thermometer. Electron. Lett..

[78] Kyuma K., Tai S., Sawada T., Nunoshita M. (1982). Fiber-optic instrument for temperature measurement. IEEE Trans. Microw. Theory Tech..

[79] Betta G., Pietrosanto A. (2000). An intrinsic fiber optic temperature sensor. IEEE Trans. Instrum. Meas..

[80] Hernandez D., Olalde G., Beck A., Milcent E. (1995). Bicolor pyroreflectometer using an optical fiber probe. Rev. Sci. Instrum..

[81] Müller B., Renz U. (2001). Development of a fast fiber-optic two-color pyrometer for the temperature measurement of surfaces with varying emissivities. Rev. Sci. Instrum..

[82] Da Silva Marques R., Prado A.R., Da Costa Antunes P.F., De Brito André P.S., Ribeiro M.R.N., Frizera-Neto A., Pontes M.J. (2015). Corrosion resistant FBG-based quasi-distributed sensor for crude oil tank dynamic temperature profile monitoring. Sensors.

[83] Chu C.-S., Lo Y.-L. (2007). A plastic optical fiber sensor for the dual sensing of temperature and oxygen. IEEE Photonics Technol. Lett..

[84] Minardo A., Bernini R., Zeni L. (2013). Distributed temperature sensing in polymer optical fiber by BOFDA. IEEE Photonics Technol. Lett..

[85] Tapetado A., Diaz-Alvarez J., Miguelez H., Vazquez C. (2016). Fiber-optic pyrometer for very localized temperature measurements in a turning process. IEEE J. Sel. Top. Quantum Electron..

[86] Murtaza G., Senior J. (1993). Referencing strategies for intensity modulated optical fibre sensors: A review. Opt. Laser Technol..

[87] Garcia C.V., Montalvo J., Lallana P.C. (2005). Radio-frequency ring resonators for self-referencing fiber-optic intensity sensors. Opt. Eng..

[88] Tapetado A., Pinzon P.J., Zubia J., Vazquez C., Vazquez M.C., Moraleda A.T., Castillo P.J.P. (2015). Polymer optical fiber temperature sensor with dual-wavelength compensation of power fluctuations. J. Light. Technol..

[89] Leal-Junior A., Frizera-Neto A., Marques C., Pontes M.J. (2018). A polymer optical fiber temperature sensor based on material features. Sensors.

[90] Pérez-Ocón F., Rubino M., Abril J., Casanova P., Martinez J. (2006). Fiber-optic liquid-level continuous gauge. Sens. Actuators A Phys..

[91] Raatikainen P., Kassamakov I., Kakanakov R., Luukkala M. (1997). Fiber-optic liquid-level sensor. Sens. Actuators A Phys..

[92] Pérez-Castellanos J.L., Montero D.S., Vázquez C., Zahr-Viñuela J., González M. (2015). Photo-thermo-mechanical behaviour under quasi-static tensile conditions of a PMMA-core optical fibre. Strain.

[93] Matsuura M., Sato J. (2014). Bidirectional Radio-over-fiber systems using double-clad fibers for optically powered remote antenna units. IEEE Photonics J..

[94] Matsuura M., Furugori H., Sato J. (2015). 60 W power-over-fiber feed using double-clad fibers for radio-over-fiber systems with optically powered remote antenna units. Opt. Lett..

[95] RLH Industries Inc. https://www.fiberopticlink.com/product/fiber-optic-isolation-systems/power-solutions-for-fiber-optic-isolation-systems/power-over-fiber-system-pof/#tab-id-5.

[96] MH GoPower. http://www.mhgopower.com/laser_pof_concept.html.

[97] Lopez-Cardona J.D., Sánchez Montero D., Vazquez C. (2019). Smart remote nodes fed by power over fiber in internet of things applications. IEEE Sens. J..

[98] Kuzyk M.G. (2007). Polymer Fiber Optics: Materials, Physics, and Applications.

[99] López-Cardona D., Sánchez Montero D., Pinzón-Castillo P.J., Vázquez C. GIPOF-based power delivery systems. Proceedings of the 25th International Conference on Plastic Optical Fibres (POF).

[100] Vázquez C., Montero D.S., Pinzón P.J., López-Cardona J.D., Contreras P., Tapetado A. Integration of power over fiber on RoF systems in different scenarios. Proceedings of the SPIE 10128 OPTO, Broadband Access Communication Technologies XI.

[101] Koonen A.M.J., Tangdiongga E.E. (2013). Photonic home area networks. J. Light. Technol..

[102] Al-Zubaidi F.M.A., Montero D.S., Vazquez C. (2021). SI-POF supporting power-over-fiber in multi-Gbit/s transmission for in-home networks. J. Light. Technol..

[103] Fahad Al-Zubaidi M.A., Montero D.S., López A., Zubia J., Vázquez C. Investigation of power over fiber impact on gigabit data transmission in SI-POF. Proceedings of the International Conference on Plastic Optical Fiber (ICPOF 2019).

[104] Pinzon P.J., Pérez I., Vazquez C., Vazquez M.C., Castillo P.J.P. (2016). Visible WDM system for real-time multi-Gb/s bidirectional transmission over 50-m SI-POF. IEEE Photonics Technol. Lett..

[105] Pinzón P.J., Moreno J., Vázquez C. Energy efficiency of a WDM link at multi-Gbit/s over plastic optical fiber. Proceedings of the National Symposium International Scientific Radio Union (URSI).

[106] Flipsen T.A.C., Steendam R., Pennings A.J., Hadziioannou G. (1996). A novel thermoset polymer optical fiber. Adv. Mater..

[107] López A., Jiang X., Losada M.A., Mateo J., Richards D., Madamopoulos N., Antoniades N. Temperature sensitivity of POF links for avionics applications. Proceedings of the 19th International Conference on Transparent Optical Networks (ICTON).

[108] Lopez A., Losada M.A., Mateo J., Jiang X., Richards D.H., Antoniades N., Losada A. (2018). Transmission performance of plastic optical fibers designed for avionics platforms. J. Light. Technol..

[109] Losada M.A., Domínguez-Chapman F.A., Mateo J., López A., Zubia J. Influence of termination on connector loss for plastic optical fibres. Proceedings of the 16th International Conference on Transparent Optical Networks (ICTON).

[110] Grivas E., Syvridis D., Friedrich G. (2009). Influence of connectors on the performance of a VCSEL-based standard step-index POF link. IEEE Photonics Technol. Lett..

[111] Kobayashi S., Horiguchi K., Hyakutake Y., Sugihara O. (2017). Evaluation of modal power distribution of automotive optical gigabit ethernet connections. J. Light. Technol..

[112] Richards D.H., Losada M.A., Antoniades N., Lopez A., Mateo J., Jiang X., Madamopoulos N. (2012). Modeling methodology for engineering SI-POF and connectors in an avionics system. J. Light. Technol..

[113] Mateo J., Losada M.A., Zubia J. (2009). Frequency response in step index plastic optical fibers obtained from the generalized power flow equation. Opt. Express.

[114] Esteban A., Losada M.A., Mateo J., Antoniades N., Lopez A., Zubia J. Effects of connectors in SI-POFs transmission properties studied in a matrix propagation framework. Proceedings of the International Conference on Plastic Optical Fiber (ICPOF 2011).

[115] Mateo J., Losada M.A., López A. (2015). POF misalignment model based on the calculation of the radiation pattern using the Hankel transform. Opt. Express.

[116] López A., Losada M.A., Richards D., Mateo J., Jiang X., Antoniades N. Statistical approach for modeling connectors in SI-POF avionics systems. Proceedings of the 21st International Conference on Transparent Optical Networks (ICTON).

[117] López A., Losada A., Mateo J., Zubia J. On the variability of launching and detection in POF transmission systems. Proceedings of the 20th International Conference on Transparent Optical Networks (ICTON).

[118] Zanon M.C., Silva V.N.H., Barbero A.P.L., Ribeiro R.M. (2018). Practical splicing of poly-methyl-methacrylate plastic optical fibers. Appl. Opt..

[119] Trouillard G., Cerutti R., Pruneau-Godmaire X., Leblanc F., Zivojinovic P., Weynant E. New mechanical splices for single and ribbon fibres. Proceedings of the 2010 Avionics, Fiber-Optics and Photonics Technology Conference.

